# Protective Effect of Parsley Juice (*Petroselinum crispum*, Apiaceae) against Cadmium Deleterious Changes in the Developed Albino Mice Newborns (*Mus musculus*) Brain

**DOI:** 10.1155/2016/2646840

**Published:** 2016-02-07

**Authors:** Ahmed A. Allam, Salah N. Maodaa, Rasha Abo-Eleneen, Jamaan Ajarem

**Affiliations:** ^1^Department of Zoology, College of Science, King Saud University, Riyadh 11451, Saudi Arabia; ^2^Department of Zoology, Faculty of Science, Beni-Suef University, Beni-Suef 62511, Egypt

## Abstract

Parsley was used as a probe of the current experiment to prevent the behavioral, morphological and biochemical changes in the newborn brain following the administration of cadmium (Cd) to the pregnant mice. The nonanesthetized pregnant mice were given daily parsley juice (*Petroselinum crispum*) at doses of 20 mg/kg and 10 mg/kg. Pregnant mothers were given Cd at a dose of 30 mg/kg divided into 3 equal times. The newborns have been divided into 6 groups: Group A, mothers did not take treatment; Groups B and C, mothers were treated with low and high dose of parsley, respectively; Group D, mothers were treated only with Cd (perinatal intoxication); Groups E and F, mothers were treated with Cd doses and protected by low and high doses of parsley, respectively. Light microscopy showed that Cd-induced neuronal degeneration by chromatolysis and pyknosis in the brain regions. The low dose of parsley 10 g/kg/day exhibited significant effects in neutralizing and reducing the deleterious changes due to Cd exposure during pregnancy on the behavioral activities, neurotransmitters, oxidative stress, and brain neurons morphology of the mice newborns.

## 1. Background

Cadmium (Cd) exposure produces severe toxicity in multiple organ tissues because it produces oxidative stress, disrupts aquaporins, and interferes with functions of essential cations such as zinc and magnesium [1]. Cd causes high risks to the young, as exposures early in life during development [2]. It is classified as one of the most toxic and carcinogenic metals [3]. It was reported as a serious industrial and environmental pollutant and may cause serious health hazards to humans and animals [4]. Many sources of Cd exposure for humans and animals could be from several industries (such as petroleum mining, metal plating, pigments, plastics, batteries, toys, and alloy), cigarette smoking, or dietary consumption [5]. Exposure to Cd may cause lesions in many organs such as central nervous system (CNS), liver, kidney, and testis [1, 3, 6].

The long-term changes in neurobehaviors such as alterations in attention and memory as well as in the psychomotor and vasomotor functioning and speed in workers are due to Cd exposure [7]. Moreover, rat studies have observed increased aggressive and anxiety-like behaviors, impaired learning and memory processes, and changes in the development of the visual system [8]. Some studies on Cd toxicity have found an association with behavioral disturbances and cholinergic neurotransmission since an increase or a decrease in the acetylcholine esterase activity was verified in both animal models and humans that showed behavioral impairments after exposure to Cd [9]. This enzyme hydrolyses the neurotransmitter acetylcholine in the synaptic cleft of cholinergic synapses and neuromuscular junctions [10]. Alterations in the acetylcholine activity in various diseases and poisonings suggest that this enzyme could be an important physiological and pathological parameter [11]. In addition, maternal Cd exposure during pregnancy induced fetal growth restriction [12]. Nevertheless, the molecular mechanism for Cd-induced development toxicity remains obscure.

Cd has well-documented teratogen and embryotoxic effects in a large variety of species, including man [13]. Cd is more toxic to newborns and young rats than to adult [14]. This metal accumulates in the brain of developing and adult rats [11] leading to brain intracellular accumulation, cellular dysfunction, and cerebral edema. Also, it can affect the degree and balance of excitation-inhibition in synaptic neurotransmission as well as the antioxidant levels in animal brain [15]. Cd embryotoxicity is partly due to oxidative DNA damage associated with increased producing of oxygen reactive species (ROS) and decreased antioxidant enzyme levels, and the interaction of Cd with the enzymes that repair damaged DNA [16]. Many culinary herbs (e.g., parsley) have been shown to function as natural antioxidants [17].

Parsley (*Petroselinum crispum*, Apiaceae) is an annual herb which is important dietary source of vitamins and essential metals. Its supplementation at sufficient levels can promote the levels of the vitamins and essential metals in the human body, which in turn can decrease the risks of Cd toxicity [18]. Phytochemical screening of parsley has revealed the presence of some compounds such as flavonoids [19], carotenoids [20], ascorbic acid [21], and tocopherol [22]. These components of fresh parsley leaf scavenge superoxide anion in vitro and hydroxyl radical in addition to protecting against ascorbic acid-induced membrane oxidation [19], where lipid oxidation is a major cause of food quality deterioration. Supplementation of diets with fresh parsley leaf can increase antioxidant capacity of rat plasma and decrease oxidative stress in humans [23]. Similarly, aqueous and ethanol extracts of fresh parsley leaf strongly inhibit linoleic acid oxidation and lipid oxidation [24].

Parsley is reported as a good source of antioxidant which may prevent Cd toxicity and teratogenicity. Also, it is one of the most used medicinal plants to treat arterial hypertension [25], diabetes, cardiac [25], and renal diseases [26]. Moreover, in experimental studies, it has been reported that this herb has strong diuretic [27], antihyperglycemic [28], antihyperlipidemic, anticoagulant [29], antioxidant [30], antimicrobial [31], and laxative activities [32]. Alcoholic extract of parsley has a protective effect against toxicity induced by sodium valproate (SVP) in male rats [33]. Parsley leaf was used for treatment of constipation, jaundice, colic, flatulence edema, and rheumatism. It was used as an aphrodisiac, improved productive performance in broiler, antimicrobial, antianemia, hemorrhagic, anticoagulant, antihyperlipidemic, antihepatotoxic, and laxative [32, 33]. It was used to treat eczema, knee, ache, impotence, and bleed [34]. However, according to our knowledge, no investigations have been reported in the literature on the protective effect of parsley against Cd teratogenicity.

In the present work we investigated the hypothesis of the protective effect of parsley juice against Cd intoxication during pregnancy and lactation periods in albino mice newborns. Behavioral and motor performances of newborns have been investigated. Also, we have evaluated the effects of this metal on oxidative stress, neurotransmitter activities, and brain structures of the newborns.

## 2. Methods

### 2.1. Chemicals

The current cadmium chloride (CdCL_2_) and other chemicals have been purchased from Sigma Company (St. Louis, MO, USA).

### 2.2. Parsley Juice Preparation

*Petroselinum crispum* (Mill.) Nym. ex A.W. Hill from the family Apiaceae (alt. Umbelliferae) is commonly known as parsley. The origin of parsley is from Mediterranean region, but today it is cultivated wherever of the world. Botanic identification was performed by taxonomist in the Department of Botany and Microbiology, Collage of Sciences, King Saud University, Riyadh, Saudi Arabia. The plain leaf of parsley type was daily collected from vegetable market in Riyadh (Saudi Arabia) and was carefully washed under tap water. The fresh parsley juice was prepared daily using a vegetable juices machine. Two concentrations of the juice were prepared: the first is 10% juice, that is, 10 g parsley squash in 100 mL drinking water. The second is 5% juice, that is, 5 g parsley squash in 100 mL drinking water. The prepared juice has been filtered using a filter paper after preparation and before drinking by the animals to remove fibers and other insoluble materials.

### 2.3. Ethics Statement

All the experimental protocols and investigations were approved and complied with the Guide of Laboratory Animals Use and Care which have been published by the United States of America National Institutes of Health (NIH Publication number 85–23, revised 1996) and have been approved by the Animal Experimentation Ethics Committee at the King Saud University (Permit number: PT 983).

### 2.4. Animals and Dosing Schedule

The current study used a total of 45 albino mice (*Mus musculus*), 15 mature males and 30 mature virgin females (weighing 30–35 g), collected from the animal house in the College of Pharmacy, King Saud University. The animals were housed in mouse cages (1 animal/cage) under pathogen-free and healthy conditions. Males and females lived at 22–25°C on a light/dark cycle (12 : 12 h), in addition to provided water and food* ad libitum*. Mating was done between proesterous females and males overnight by housing of one female with one male in special cages used for mating (stainless steel wire cages). Vaginal plug deposition at morning determined the day zero of gestation. Parsley juice was orally administered daily to nonanesthetized parsley treated groups by gastric intubation at a dose of 20 g/kg/day and 10 g/kg/day from day zero of gestation till postnatal day 30.

Totally, 30 mg/kg of CdCl_2_ was dissolved in 30 mL saline and intraperitoneally injected to partially anesthetized Cd treated groups on three stages: at day 7 of gestation, postnatal day 1, and postnatal day 15 (10 mg/kg every time).

The mothers were labeled and divided into 6 groups as follows:

 Group A: pregnant mice were given tap water orally and saline intraperitoneally (did not take treatment as control group).
 Group B: pregnant mice were given 5% parsley juice orally and saline intraperitoneally (*5% parsley group*).
 Group C: pregnant mice were given 10% parsley juice orally and saline intraperitoneally (*10% parsley group*).
 Group D: pregnant mice were given tab water orally and Cd doses intraperitoneally (*Cd intoxicated group*).
 Group E: pregnant mice were given 5% parsley juice orally and Cd doses intraperitoneally (*5% parsley-Cd group*).
 Group F: pregnant mice were given 10% parsley juice orally and Cd doses intraperitoneally (*10% parsley-Cd group*).

### 2.5. Newborns Body Weights Assessment during Lactation Period

A physical developmental landmark like body weight is a useful indicator of the development through the entire lactation period. Thus, the newborns were weighed every day from postnatal day 1 until day 28.

### 2.6. Cd Estimation Assay

The analytical determination of Cd in the brain was carried out according to Shah et al. [35] using Inductively Coupled Plasma Mass Spectrometer (ICP-MS) under operation conditions of the instruments shown in Table (S1) (see Supplementary Material available online at http://dx.doi.org/10.1155/2016/2646840).

### 2.7. Behavioral Assays

Ten meal mice pups from each group (first generation) were investigated in the current study at postnatal day 30 (postweaning period). Each newborn was used only in one test. For tests conduction, the pups were brought into investigation room of dim red light used for this purpose (25°C). The tests were done blindly by one experimenter according to Ajarem and Ahmad [36].

#### 2.7.1. Cage Activity Assay

The Ugo Basile 47420-Activity Cage was used to record spontaneous coordinate activity in mice and variation of this activity in time either horizontal or vertical movements. This test was performed for 3 min/animal.

#### 2.7.2. Grip-Strength Meter Assay

The Ugo Basile 47200 Grip-Strength Meter suitable for mice automatically measures Grip Strength (i.e., peak force and time resistance) of the forelimbs in mice. The aim was to assess forelimbs muscle strength. Each animal was tested three times and the peak force of each mouse was recorded. The mean of three values of each mouse was recorded.

#### 2.7.3. Rota-Rod Assay

The Ugo Basile rota-rod instrument has been used to estimate the balance ability in the investigated animals. The newborns were placed on a horizontally oriented rod which mechanically rotates at 10 ×g. The newborns innately will try to stay on the rota-rod (rotating rod) and will try to avoid falling down on the instrument sensor. The length of time that the tested newborns stay on the rota-rod was used as a measure of their coordination, physical condition, balance, and motor activity.

### 2.8. Biochemical Assays

Six newborns from each group were anesthetized by light ether and sacrificed by decapitation at postnatal day 30. The brain was dissected and 0.5 g tissue was homogenized in 5 mL of cold 0.1 M HClO4 containing 0.05% EDTA. The homogenate was centrifuged at 1000 ×g for 10 min at 4°C and the clear supernatant was collected in a microfuge tube (0.5 mL each) and was stored at −40°C until used.

#### 2.8.1. Dopamine and Serotonin Determination Assay

The levels of neurotransmitters serotonin or dopamine were estimated in the brain using the modified method of Eghwrudjakpor et al. [37] as mentioned by Abu-Taweel et al. [38].

#### 2.8.2. Acetylcholine Determination Assay

The estimation of acetylcholine was done according to the method of Ichikawa et al. [39].

#### 2.8.3. Lipid Peroxidation Assay

Lipid peroxidation was estimated by assessment of thiobarbituric acid-reactive substances (TBARS) using the method of Preuss et al. [40].

#### 2.8.4. Glutathione (GSH) Assay

The reduced glutathione content was estimated using the modified method of Beutler et al. [41] as reported in Allam et al. [42].

#### 2.8.5. Peroxidase Activity Determination Assay

Peroxidase enzyme activity was estimated using the procedure of Kar and Mishra [43].

### 2.9. Histological Studies Using Light Microscopy

Four pups of each group have been anesthetized using light ether and decapitated at postnatal day 30 followed by dissection. Histological preparations of the left loop of cerebellum, cerebral cortex, and medulla oblongata were done as mentioned by Allam et al. [42]. The use of Haematoxylin and Eosin stains for the paraffin sections was done according to the method of Mallory [44].

### 2.10. Statistical Analysis Assays

The current study data has been analyzed using the software program Statistical Package for the Social Sciences (SPSS for Windows version 11.0; SPSS Inc., Chicago). The comparative analyses were conducted by using the general linear models procedure (SPSS, Inc.). The data has been tested by one-way and two-way analysis of variance (ANOVA) followed by LSD computations to compare various groups with each other. Results were expressed as mean ± SD. The level of significance was expressed as very highly significant at ^*∗∗∗*^ *P* < 0.001, highly significant at ^*∗∗*^ *P* < 0.01, and significant at ^*∗*^ *P* < 0.05.

## 3. Results

### 3.1. General Observations

**Figure 1:**
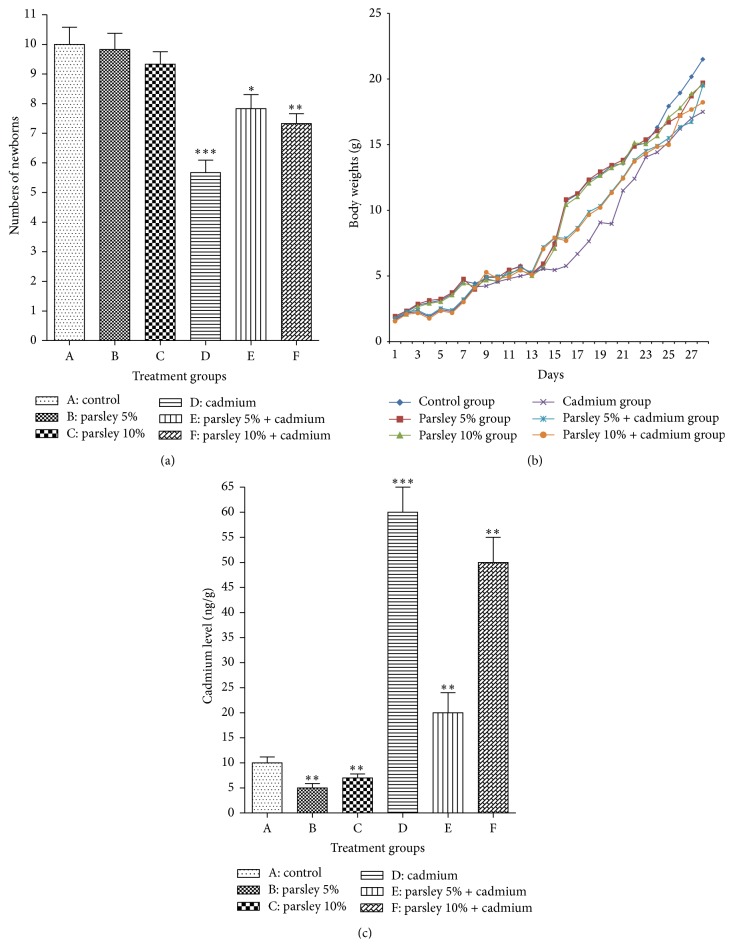
Postnatal developmental observations. (a) The number of alive delivered newborns (litter size) of each group at postnatal day 1. (b) The newborns body weights of each group from postnatal day 1 to day 28. (c) The Cd concentration in the brain tissues of the newborns of each group at postnatal day 30. Data are expressed as mean ± SE (*N* = 6; ^*∗*^ *P* < 0.05, ^*∗∗*^ *P* < 0.01, and ^*∗∗∗*^ *P* < 0.001, significantly different from the control group).

The signals of toxicity due to Cd exposure were noticed postnatally in group D mothers represented by weakness and ataxia of hindlimb muscles causing alteration in maternal behavior, so their newborns suffered from bad lactation and consequently malnutrition. These signals of toxicity were reduced in groups E and F and especially group E due to parsley supplementation. The insignificant difference in letters size was observed in groups B, C, and E while highly significant and very highly significant reduction were detected in groups F and D (Figure 1(a)) compared to group A. The body weights of the pups of the present experimental groups were varied from postnatal day 1 to day 28 (Figure 1(b)). The pups of groups D, E, and F suffered from a noticeable decrease in the body weight gain especially group D. The pups of group E showed marked increase in their body weight gain by the third week of age to become near the values of normal pups.

The Cd concentration in the brain tissues was estimated in the brain tissues of the newborns.  Figure 1(c) showed the Cd bioaccumulation in the brain of groups B and C was highly significantly reduced while in group D it was very highly significantly elevated and in groups E and F it was highly significantly elevated compared to group A. The current results showed that parsley significantly reduces the Cd concentration in pup's brain of parsley treated group.

### 3.2. Behavioral Investigations

**Figure 2:**
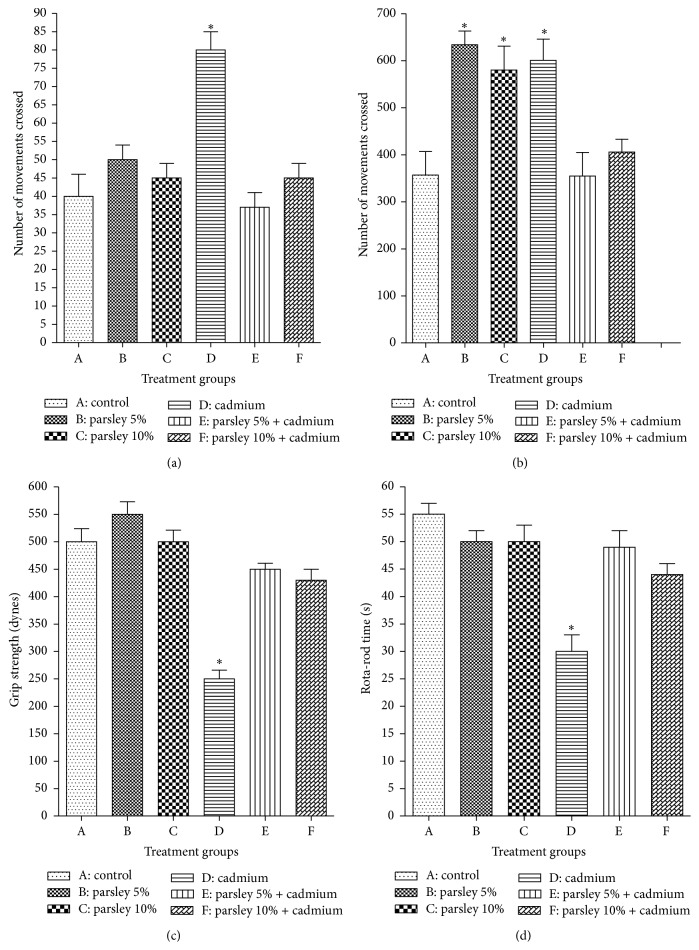
Behavioral investigations of the newborns of each group at D 30. (a) Vertical movements, (b) horizontal movements, (c) grip strength records for the forelimb, and (d) rota-rod records. Data are expressed as mean ± SE (*N* = 6; ^*∗*^ *P* < 0.05, significantly different from the control group).

In the activity cage and in comparison with the newborns of group A, the newborns of group D showed a significant elevation in the vertical and horizontal movement in comparison with group A. Contrary to the vertical movement, the newborns of groups B and C showed significant increase in the value of horizontal movement. Despite Cd and parsley treatment, the newborns of groups E and F displayed vertical and horizontal movement values near the values of normal newborns (Figures 2(a) and 2(b)).

The forelimb muscles of the newborns of groups A, B, and C recorded relatively similar beaks in the grip strength examination scores. While the recorded beaks of group D newborns appeared significantly small, the beaks achieved by the newborns of groups E and F showed significant improvements (Figure 2(c)).

In rotator test and in comparison with the newborns of group A, the time of group D pups on the rod was significantly small. The staying times of the newborns of groups B, C, E, and F on the rod were less than the time of group A but the difference was not significant (Figure 2(d)).

### 3.3. Biochemical Studies

#### 3.3.1. Neurotransmitters

**Figure 3:**
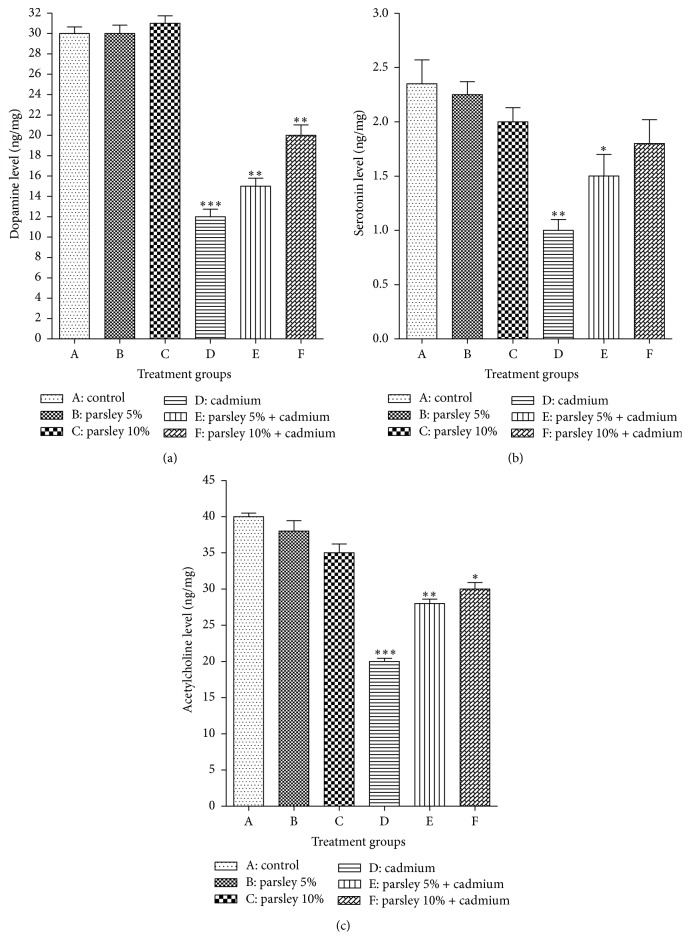
Extracellular neurotransmitters concentration in the newborns brain tissues at postnatal day 30. (a) Dopamine, (b) serotonin, and (c) acetylcholine. Data are expressed as mean ± SE (*N* = 6; ^*∗*^ *P* < 0.05, ^*∗∗*^ *P* < 0.01, and ^*∗∗∗*^ *P* < 0.001, significantly different from the control group).

In groups A, B, and C, the dopamine concentration in the brain appeared nearly similar with little elevation in group C. In comparison with group A, very highly significant depletion of dopamine concentrations has been detected in group D (*P* < 0.001) and highly significant reduction in groups E and F (*P* < 0.01) as in Figure 3(a).

No significant differences in the brain-serotonin concentration were detected between groups A, B, C, and F while highly significant reduction (*P* < 0.01) and significant decrease (*P* < 0.05) have been detected in groups D and E, respectively (Figure 3(b)).

The present acetylcholine concentrations showed similar results to dopamine. In groups A, B, and C, its concentration did not have any insignificant differences while it displayed very highly significant (*P* < 0.001), highly significant (*P* < 0.01), and significant (*P* < 0.05) depletion in groups D, E, and F, respectively (Figure 3(c)).

#### 3.3.2. Oxidative Stress

**Figure 4:**
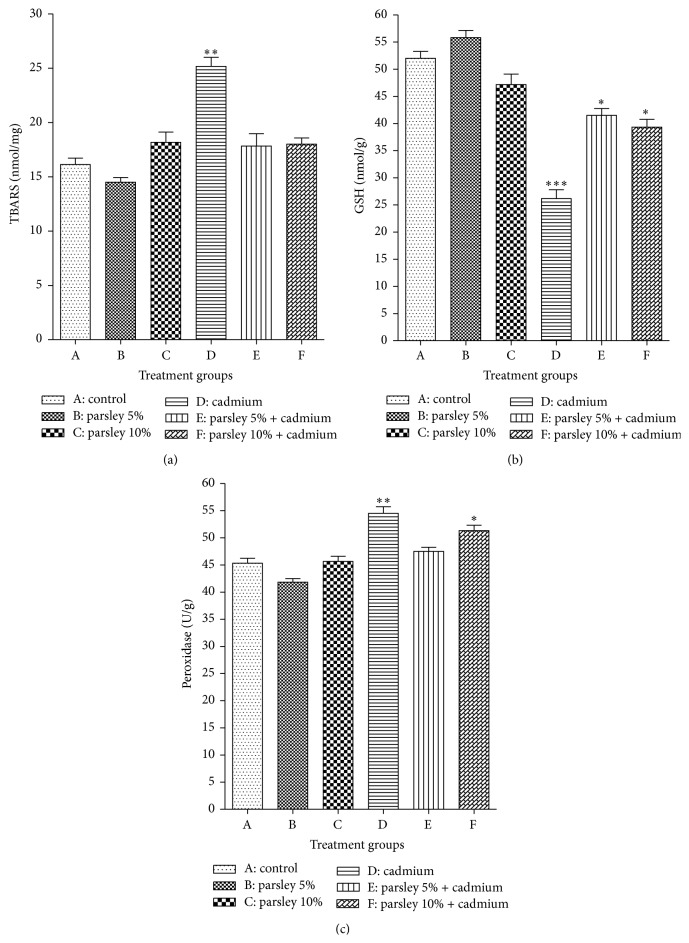
Oxidative stress parameters in the brain tissues of the newborns of each group at postnatal day 30. (a) TBARS, (b) GSH, and (c) peroxidase. Data are expressed as mean ± SE (*N* = 6; ^*∗*^ *P* < 0.05, ^*∗∗*^ *P* < 0.01, and ^*∗∗∗*^ *P* < 0.001, significantly different from the control group).

In comparison with control newborns, the brain lipid peroxidation was insignificantly reduced in group B while insignificant increase was observed in group C (Figure 4(a)). The elevation of TBARS was highly significant (*P* < 0.01) in group D and insignificant (*P* > 0.05) in groups E and F (Figure 4(a)).

Cd exposure produced noticeable reduction in GSH content (*P* < 0.001) in group D (~50%). Improvement was observed in groups E and F although the difference between them and control group was still significant (*P* < 0.05) according to Figure 4(b). Insignificant difference (*P* > 0.05) was observed between groups A, B, and C (Figure 4(b)).

Peroxidase activity showed fluctuations in the experiment groups. No significant difference was observed between groups A and C while an insignificant reduction was noticed in group B. Cd exposed groups showed significant increase of peroxidase activity in both groups D and F while the increase was insignificant (*P* > 0.05) in group E (Figure 4(c)). The maximal elevation of peroxidase activity was observed in group D (*P* < 0.01).

### 3.4. Brain Histoarchitecture Changes

**Figure 5:**
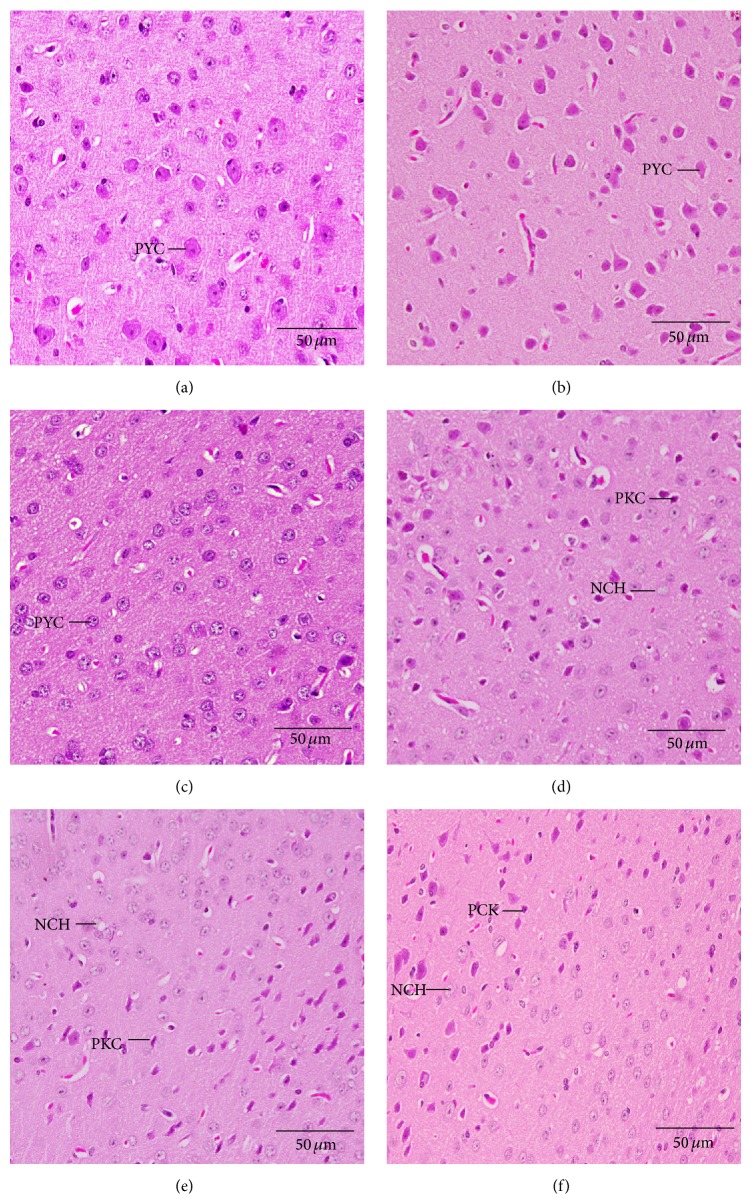
Histological changes in the cerebral cortex of the newborns at postnatal day 30 showing pyramidal neurons (PYC), degenerated pyramidal cells (PKC), and neurocyte chromatolysis (NCH). (a) Control group, (b) parsley 5% group, (c) parsley 10%, (d) cadmium inoculated group, (e) cadmium inoculated group + parsley 5%, and (f) cadmium inoculated group + parsley 5% (H&E stain).

At postnatal day 30, the normal pyramidal neurons exhibited their general characteristic shape. The nuclei of these cells were rounded, large, and centrally located (Figure 5). The normal cerebral cortex cells had pyramidal or spherical perikaryon with large nuclei where the cells were arranged in a uniform pattern (Figures 5(a), 5(b), and 5(c)). These neurons seemed more developed if we move toward the white matter. The cerebral investigation of many sections in the Cd exposed groups showed some pathological cases. In group D, chromatolysis and pyknosis have been observed in the pyramidal neurons. In groups E and F, parsley juice showed significant neuronal protection through reducing the rate of chromatolysis and pyknosis (Figures 5(d), 5(e), and 5(f)).

**Figure 6:**
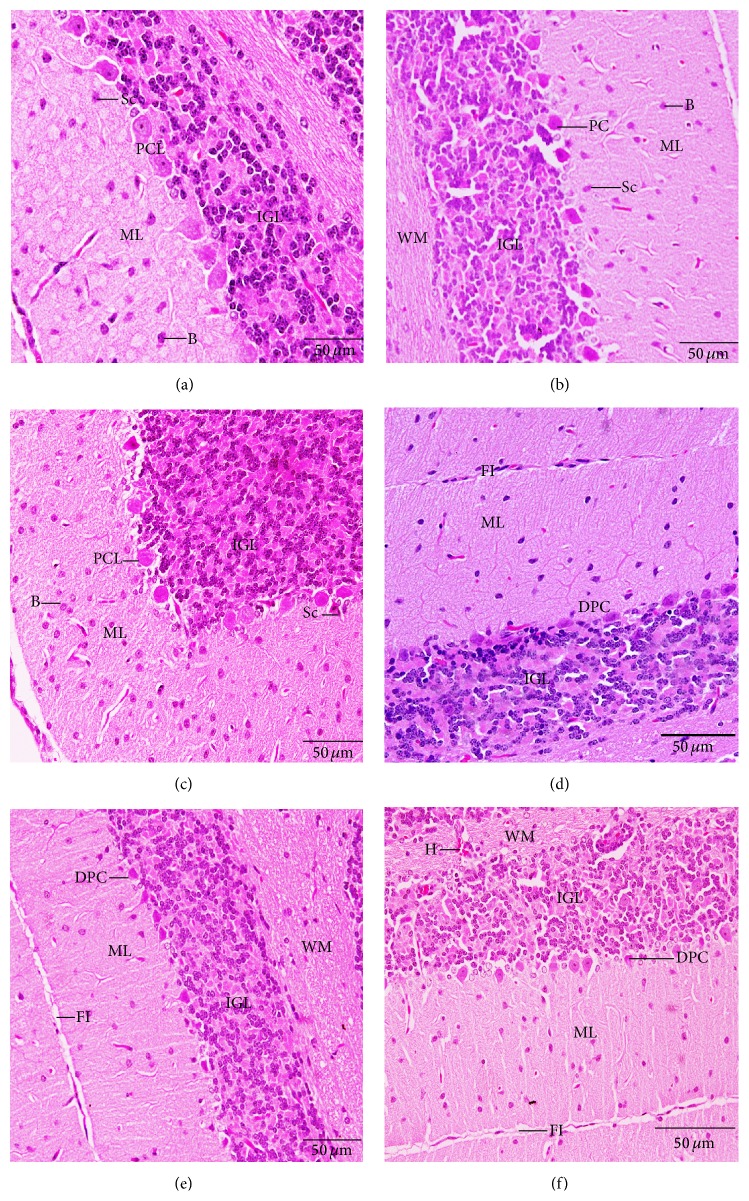
Histological changes in the cerebellar cortex of the newborns at postnatal day 30 showing Purkinje cell (PC), Purkinje cell layer (PCL), degenerated Purkinje cell (DPC), fissure (FI), hemorrhage (H), internal granular layer (IGL), molecular layer (ML), and white matter (WM). (a) Control group, (b) parsley 5% group, (c) parsley 10%, (d) cadmium inoculated group, (e) cadmium inoculated group + parsley 5%, and (f) cadmium inoculated group + parsley 5% (H&E stain).

In the cerebellum, the fold layers (molecular, Purkinje cells, and internal granular) have become completely mature and the external granular layer disappeared completely (Figure 6). The neuronal density in the molecular layer of normal and parsley treated groups was the highest compared to Cd treated groups. The normal Purkinje cells had a pear-shaped perikaryon and large nucleus and were arranged in a single row. The lateral processes disappeared and the apical processes formed the permanent dendritic tree (Figure 6). In Cd treated groups, some pyknotic and degenerated Purkinje cells were observed and some were more spindle-shaped and small. These numbers of degenerated Purkinje neurons reduced in groups E and F (Figures 6(d), 6(e), and 6(f)). Variations have been observed in the folds size of the groups where small folds appeared in group D.

**Figure 7:**
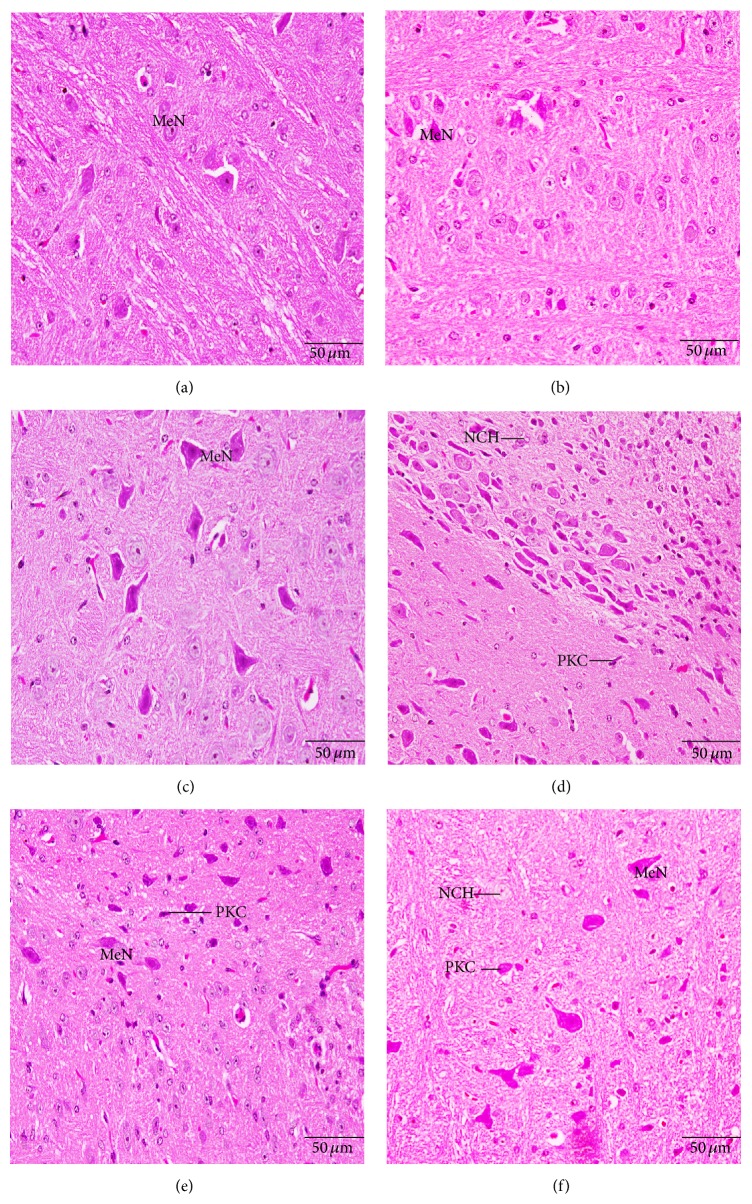
Histological changes in the medulla oblongata of the newborns at postnatal day 30 showing medulla neurons (MeN), degenerated medullary cells (PKC), and neurocyte chromatolysis (NCH). (a) Control group, (b) parsley 5% group, (c) parsley 10%, (d) cadmium inoculated group, (e) cadmium inoculated group + parsley 5%, and (f) cadmium inoculated group + parsley 5% (H&E stain).

The normal medulla neurons appeared to be large in size, polygonal, and varied in shape and had round nuclei (Figures 7(a), 7(b), and 7(c)). In Cd treated group, most of medulla neurons appeared small and pyknotic (Figure 6(d)). Cd-parsley treated groups medulla neurons showed improvement (Figures 6(e) and 6(f)).

## 4. Discussion

The current study has been designed to investigate the protective role of parsley juice against Cd teratogenicity in albino mouse newborns. These results proposed that the intake of parsley may improve the malformations due to exposure of female pregnant mice to Cd. The effect of daily supplementation of the two different doses of parsley on the deleterious changes of Cd in litter size, pups body weight, behavioral activities, brain neurotransmitters, brain oxidative stress, brain Cd concentration, and brain histoarchitecture was discussed in the current study. The small litter size of the nonprotected Cd treated group shows the hazardous effect of Cd on pregnancy. Antonio et al. [14] recorded that prenatal Cd exposures induce fetal resorption and abortion. Cd passes through placenta and is dispensed in the embryos tissues during pregnancy [11]. The high accumulation of Cd in brain tissues of Cd treated groups due to prenatal and postnatal passing through placenta and mother's milk to its newborns as reported early by Shaheed et al. [45]. The low concentration of Cd in the pup's brain tissue in groups E and F may be explained by the ability of the mothers of these two groups to get off the administrated Cd due to the ingested parsley.

The current results reported that the Cd exposed groups showed reeducation in the pup's body weights as reported in earlier studies. The reasons this weight loss may be prenatal due to intrauterine Cd exposures that produce growth deficiency for the developed fetus or postnatal as maternal Cd exposure causes bad lactation due to maternal bad behaviors caused also by Cd and consequently leads to postnatal malnutrition fort the neonates [45]. In addition, Friedman et al. [46] confirmed the main reason for the postnatal pup's weight loss because they culminate from maternal behaviors alterations in Cd exposed groups as well as a decrease in the lactation index. Pups body weights are the most sensitive indicator of developmental toxicity [47]. The results of groups E and F showed that parsley juice has an observable protective effect against Cd accumulation and prenatal effects, especially the low dose. These may be due to the significant effect of parsley in the excretion of heavy metals such as Cd from mother's bodies so the complications of Cd toxicity reduced and disappeared in the pups of parsley ingested groups [27].

The present levels of the neurotransmitters were depleted significantly by Cd treatment in the brain tissue of the Cd exposed groups. The inhibition of dopamine, serotonin, and acetylcholine occurs in depressing the hyperexcitability of brain neurons as what appeared by the poor performance of the treated pups in the current behavioral examinations [3]. Recently, a growing body of research has focused on the participation of serotonin in the neurochemical mechanisms of cognition and especially of learning and memory. Potential toxic mechanisms of action for Cd may include disruption in serotonergic neurotransmission through disturbed levels of neurotransmitters in the brain [3]. It was mentioned before that Cd toxicity disrupts the action of the acetylcholine esterase activity, so the sensorimotor performances of the newborns will be affected [9]. The disturbances in the levels of current neurotransmitters levels in the toxicity exposed groups may be due to the impact of Cd on the neurons functions [3], while the improvements in the parsley juice ingested groups may be because the ability of this component to limit the Cd impacts the neurons.

Although groups D, E, and F pups were exposed to Cd perinatally (during gestation and lactation), group D displayed a marked elevation in the oxidative stress and a depletion of the antioxidants, compared to groups E and F. One aspect of the oxidative stress elevation was the significant increase of the lipid peroxidation in group D. Méndez-Armenta and Ríos [15] reported that the lipid peroxidation and TBARS increased after acute Cd toxicity. Glutathione is one of the most important compounds for the preserve of cell integrity because of its reducing ability and participation in the cell metabolism as well as a well sensitive reflector of the oxidative stress state in laboratory animals and human [48].

It was observed in many oxidative stress states that the reduction of GSH induces the elevation of lipid peroxidation. Therefore, GSH is an important indicator and biomarker of oxidative stress [49, 50]. Almost the level of GSH is regulated by glutathione reductase (GR) which is a NADPH dependent enzyme. Therefore the limitation in active GR and NADPH may adversely affect GSH levels [50]. Furthermore, interaction of electrophilic xenobiotics such as heavy Cd and its metabolites products with GSH forms glutathione-S conjugates which deplete GSH concentrations in the tissue cells [40, 51]. Abu-Taweel et al. [3] reported that Cd exposure depleted GSH content and elevates peroxidase enzymes activities in adult mice. Increasing of peroxidase activity may be due to the presence of generated free radicals [52]. Peroxidases are considered as one of the antioxidant enzymes which constitute a mutually supportive defense team against free radicals such as reactive oxygen species (ROS) as mentioned by Allam et al. [50].

Several mechanisms may explain the reasons of oxidative stress increasing due to heavy metals toxicity, especially the chronic toxicity. For example, the chronic Cd inhalation leads to Cd accumulation which elevates ROS and reactive nitrogen species (RNS) production by the mitochondrial respiratory system. This will exhaust the antioxidant enzymes and an imbalance of glutathione redox status will be produced [7]. At altitude concentration, ROS/RNS will damage the components of the cell including nucleic acids, proteins, amino acids, and lipids [53]. Such oxidative modifications affect several cell metabolic reactions, functions, and gene expression which in turn can cause other pathological conditions [54]. The oxidative stress leads to neuronal damage in several brain regions [40, 49, 55]. For example, neuronal loss in cerebrum impairs animal's memory [38], neuronal loss in cerebellum can have effect on balance and coordination [40], and neuronal loss in medulla oblongata and spinal cord can affect physical activity of mice [56].

Phytochemical screening of parsley has revealed the presence of some compounds such as flavonoids [19], carotenoids [20], ascorbic acid [21], and tocopherol [22]. These components of fresh parsley leaf scavenge superoxide anion in vitro and hydroxyl radical in addition to protecting against ascorbic acid-induced membrane oxidation [19]. Supplementation with parsley juice for 50 days prevent to somewhat the Cd toxicity showed significant improvement in the physical balance, coordination, motor activities, muscles strength, and brain neurotransmitters levels in Cd treated pups of groups E and F. Parsley supplement also restored GSH balance and decreased lipid peroxidation and peroxidase activity. Overall, this study demonstrated that low dose of parsley supplementation significantly improved pathological alterations in mice as reported by Zhang et al. [30]. Parsley juice components were found to be significant suppressors to H_2_O_2_ and ROS levels in brain and other tissues in mice by stimulating production of glutathione synthesis and thereby boosting cellular antioxidant defense [30]. Therefore, we suggest that parsley may be an important therapeutic tool to combat oxidative stress-associated diseases. We propose that diluted parsley juice may ameliorate Cd neurotoxicity complications in mice by its ability to neutralize free radicals and thereby prevent neuronal damage caused by oxidative stress.

## 5. Conclusion

Parsley has a protective effect against Cd neurotoxicity and teratogenicity in albino mice. Parsley juice supplementation improves the behavior of perinatally Cd intoxicated mice newborns and reduces neuronal aberrations in the brain caused by oxidative stress.

## Supplementary Material

Operation conditions of ELAN 9000 ICP-MS.
