# Supplementary material for: Protective Effect of Parsley Juice (Petroselinum crispum, Apiaceae) against Cadmium Deleterious Changes in the Developed Albino Mice Newborns (Mus musculus) Brain
Source: Oxid Med Cell Longev. 2016 Feb 7;2016:2646840. doi: 10.1155/2016/2646840 (PMC4761399; doi:10.1155/2016/2646840)
Supplement: Supplementary file 1 — Operation conditions of ELAN 9000 ICP-MS. [file 2646840.f1.pdf]

Supplementary table 1 Conditions of ELAN 9000 ICP-MS

RF power	1250 W
Nebulizer gas flow	0.92 L/min
Lens Voltage	9.25 V
Analog Stage Voltage	-1762.5 V
Pulse Stage Voltage	1050 V
Number of Replicates	3
Reading / Replicates	20
Scan Mode	Peak Hopping
Dwell Time	40 ms
Integration	1200ms
